# Multiple myeloma: changes in serum C-terminal telopeptide of collagen type I and bone-specific alkaline phosphatase can be used in daily practice to detect imminent osteolysis*

**DOI:** 10.1111/j.1600-0609.2010.01417.x

**Published:** 2010-05

**Authors:** Thomas Lund, Niels Abildgaard, Thomas L Andersen, Jean-Marie Delaisse, Torben Plesner

**Affiliations:** 1 Department of Haematology, Vejle HospitalVejle, Denmark; 2 Department of Clinical Cell Biology, IRS-CSFU, University of Southern DenmarkVejle Hospital, Vejle, Denmark; 3 Department of Haematology, Odense University HospitalOdense, Denmark

**Keywords:** multiple myeloma, bone disease, bone markers, bisphosphonates, osteolysis

## Abstract

*Objective:* Monitoring of bone disease in multiple myeloma is becoming increasingly important because bone-protecting treatment with bisphosphonate is becoming restricted after the awareness of osteonecrosis of the jaw. Despite the potential of biochemical markers of bone remodeling to monitor dynamic bone turnover, they are not used in everyday practice. Here, we investigate their usefulness to detect imminent progressive osteolysis in relapsing patients with multiple myeloma. *Methods:* In an unselected cohort of 93 patients, we measured the bone resorption markers C-terminal telopeptide of collagen type I (CTX-I), C-terminal cross-linked telopeptide of type-I collagen generated by MMPs (ICTP), N-terminal cross-linked telopeptide of type-I collagen (NTX-I), and the bone formation marker bone-specific alkaline phosphatase (bALP) monthly for 2 yr. Retrospectively, we identified 40 cases where patients had progressive disease. We investigated how the bone markers developed prior to disease progression. *Results:* We observed that CTX-I and bALP changed significantly before progressive disease were recognized. More interestingly, these changes differed depending on whether concurrent progressive osteolysis was present. In patients with progressive osteolysis, there was a large increase in bone resorption which was not compensated by increased bone formation. In contrasts, patients with stable bone disease had only a slight increase in bone resorption which was compensated by concurrent increased bone formation. By calculating a patient-specific CTX-I/bALP ratio, we quantified the risk a patient experiences if the ratio increases. *Conclusion:* By analyzing patient-specific changes in the ratio of CTX-I/bALP, we might tailor treatment with bone-protecting agents in the individual patient.

Multiple myeloma (MM) is a B-cell malignancy characterized by proliferation of monoclonal plasma cells in the bone marrow. Bone disease is detected at diagnosis by conventional radiographs of the skeleton in 80% of the patients and may cause bone pain, pathological fractures, and hypercalcemia (1). Most patients respond to initial treatment, but eventually almost all patients will have resistant relapse and die from the disease. In the last decade, many new agents have been introduced for treatment of MM. These novel agents have a striking effect on the disease, and it is now often possible to bring a patient into remission multiple times during the course of the disease (2–4). Thus, the life of a patient with MM is now commonly characterized by multiple remissions and relapses. Continuous monitoring of early signs of end organ damage is important to enable timely intervention before serious damage has occurred.

The reason for the high incidence of bone disease in MM is that the myeloma cells produce several factors that stimulate osteoclast (OC) formation and activity (5–9) and inhibit osteoblast (OB) function and bone formation (10–13). Treatment with bisphosphonate inhibits OC activity and reduce the number of new skeletal events in MM (14), and bisphosphonates are integrated into standard management of patients with MM. However, prolonged exposure to potent bisphosphonates may cause kidney damage (15) and osteonecrosis of the jaw (ONJ) (16). This has led to a more cautious and restricted use of bisphosphonates, and most international guidelines now recommend that treatment is limited to a 2-yr period for patients in remission (17, 18). However, patients in remission are still at risk of developing new osteolytic lesions. A recent guideline for monitoring patients with MM recommends that radiographs, CT-scans, or MR scans are taken only when clinically indicated after the initial staging of the patient (19). With this strategy, substantial damage may have occurred in bone before the patient becomes symptomatic and progressive bone disease is detected. Biochemical markers of bone turnover may represent an interesting alternative to evaluate the bone status of patients with myeloma. They are not harmful and are compatible with monthly monitoring. They have the potential to detect the destructive process as soon as it starts and before a lesion becomes detectable through conventional radiography. Thus, markers should allow therapeutic intervention as soon as the problem starts. Several of these markers have been used in clinical trials in patients with MM. The trials included markers of bone resorption as well as markers of bone formation because lesions reflect not only bone destruction but also impaired bone reconstruction. These bone remodeling markers have shown good correlation to histomorphometric changes (20, 21), have yielded prognostic information in newly diagnosed patients (22–29), and shown significant changes in response to treatment (30–34).

Despite their potential interest, markers of bone remodeling are not used in everyday practice, and their usefulness to detect progressive osteolysis in relapsing patients has not even been investigated. This gap appears especially important at the present time as the typical patient with MM undergoes relapses and remissions multiple times, and because it is unknown how long the patient remains protected after the discontinuation of bisphosphonate treatment.

The aim of this study was to test if some of the most promising bone markers, the bone resorption markers CTX-I, ICTP, and NTX-I and the bone formation marker bALP could yield clinically useful information in a general myeloma population and help with decision making in daily clinical practice. More specifically, we wanted to test whether the bone remodeling markers could, at a patient-specific level, provide an early warning of progressive disease and/or development of new osteolytic lesions before symptoms occur. If so, markers of bone turnover could potentially be used to tailor bisphosphonate and other relevant therapies in the individual patient. This strategy could in some patients reveal the need of reinstating anti-resorptive or anti-myeloma treatment and could in other patients reduce exposure to unnecessary bisphosphonate, thereby minimizing the risk of long-term side effects.

## Materials and methods

### Patients

An unselected cohort of patients with multiple myeloma followed at Vejle Hospital, Denmark, had the bone degradation markers CTX-I, ICTP, NTX-I and the bone formation marker bALP measured every 4 wk for 2 yr. Measurements were conducted on 93 patients (49 men/44 women). The median age of the patients was 69 yr (range: 44–90 yr), and bone markers were measured at 1233 different time points. The study was approved by the Danish Data Protection Agency and conducted according to the national ethical guidelines and the Helsinki Declaration.

Markers of bone remodeling were measured every fourth wk. The disease status of the patients was monitored using serum M-component (IgG or IgA), serum-free light kappa/lambda chains (FLC), and ionized serum calcium at least every fourth wk. Progressive disease (PD) was defined as a 25% or greater increase in serum M-component or in patients with light-chain myeloma an increase in the difference between involved and uninvolved FLC (>100 mg/L), or definite development of one or more new bone lesions, according to the International Uniform Response Criteria for Multiple Myeloma (35). In cases with PD, the patients own bone marker values at the time point when disease activity was lowest prior to the relapse, defined by nadir of M-component or FLC ratio, were compared to the patient-specific values observed when PD was detected. The median time from the lowest disease activity as reflected by the nadir of M-component or FLC to PD was 5 months. Radiographs were performed in patients with bone symptoms and in some cases with increasing values of M-component or FLC at the discretion of the treating physician.

During the study period, twenty-nine patients presented with either biochemical and/or radiological evidence of progressive disease after having been in remission. Ten patients experienced two episodes of disease progression and one patient three episodes, giving a total of 40 episodes of PD for evaluation in this study. For these 40 cases, a retrospective analysis was performed in the prospectively collected and analyzed bone markers to see whether patient-specific changes in the markers could have predicted relapse. Details of demographics and clinical parameters for patients with PD are shown in Table 1.

**Table 1:** Patient characteristics for patients with progressive disease (PD)

Number of cases/patients	40/29
Sex (male/female)	20/20
Age* (years)	66.5(55–90)
Time from SD to PD* (months)	5(2–16)
M-component type	
IgG	26
IgA	5
Light chain only	9
Bisphosphonate status	
Naive	3
Previously treated	14
Currently treated	23
Relapse no	
1st	15
2nd	10
3rd or higher	15
Relapse defined by	
Increase in paraprotein only	25
Progression in bone disease only	5
Both	10
Progression in bone disease	
Yes	15
No	11
Unknown	14

* Age and time from SD to PD are shown as median values with ranges.

Of the 40 cases of progressive disease that was observed in the study period, we identified 26 cases where radiological evaluation was conducted at the time of PD. Fifteen patient had concomitant progressive osteolysis, whereas no progression was detectable in 11 cases. Patient-specific development in the bone markers prior to PD was analyzed for the two groups to identify distinct patterns for the two groups.

In 17 patients who received novel anti-myeloma treatment, no data were available concerning the preceding period of disease progression, either because it was first-line treatment in newly diagnosed patients or because disease progression occurred before measurement of bone remodeling markers was initiated. Of the remaining 47 patients in the cohort, 15 patients had a limited number bone marker measure points, 10 patients were on continuous anti-myeloma treatment, 2 patients had smoldering myeloma only, and 5 patients were either lost to follow-up because of transfer to other hospital or were not evaluable as they were not fasting or nadir values were missing. Fifteen patients had stable disease in the observation period, but only in five of these patients, stable bone disease were verified as bone imaging did not reveal any progression in osteolysis.

### Measurements of bone degradation and bone formation markers

Blood samples were collected in the morning from fasting patients. The samples were immediately centrifuged and the serum was stored for a maximum period of 1 month prior to analysis. Urine samples were collected as fasting second void morning urine and stored in the same way. Prior to analysis, the urine was centrifuged. Samples for bALP, NTX-I, and ICTP analysis were stored at −80°C. Samples for CTX-I analysis were stored at −20°C (36). Serum CTX-I was measured by an enzyme chemiluminescence method (Roche Diagnostic, Hvidovre, Denmark). Competitive enzyme immunoassays were used to measure serum ICTP (Orion Diagnostica, Espoo, Finland) and urine NTX-I (Ostex International, Seattle, WA, USA), respectively. Serum bALP was measured with a non-competitive enzyme immunoassay technique (Quidel Corporation, San Diego, CA, USA). ICTP, NTX-I, and BAP were analyzed in duplicates. The lower detection limits were CTX-I: 0.01 μg/L, NTX-I: 20 nmol/L, ICTP: 1 μg/L, and bALP: 0.7 units/L. Urine NTX-I was normalized and expressed relative to urine creatinine.

### Evaluation of bone disease

Development of bone disease was evaluated by skeletal survey using either conventional radiography or computer tomography (CT-scan). Skeletal survey was conducted according to ‘Guidelines for the use of imaging in the management of myeloma’ (19). All images were interpreted by a senior radiologist. Only skeletal surveys done within 1 month after first sign of PD were used for further analysis (Table 1).

### Statistical analysis

Patient-specific differences in CTX-I, BAP, CTX-I/bALP ratio, ICTP, and creatinine were analyzed using a paired *t*-test. The tests were adjusted for clustering to compensate for the fact that some patients contributed with more than one dataset. NTX-I/creatinine was analyzed both using a Wilcoxon matched pair test and a paired *t*-test adjusted for clustering. Differences in progression of bone disease were analyzed using both an unpaired *t*-test adjusted for clustering and the Cox regression model. All *P*-values are two-sided and the significance level was set at *P*≤ 0.05.

## Results

### Bone markers and disease progression

**Figure 1:**
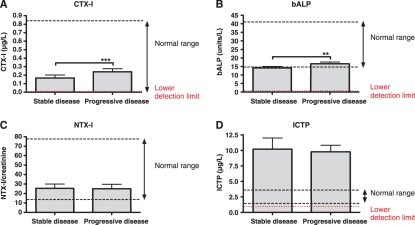
Comparison of levels of bone remodeling markers at stable disease and at first sign of progressive disease. (A) The bone resorption marker CTX-I showed a small, however, highly significant increase in patients having progressive disease. (B) Although drastically suppressed, the bone formation marker bALP increased significantly at disease progression. (C–D) Both resorption markers NTX-I and ICTP remained unchanged. Results are shown as mean ± SEM of 37 (C) and 40 (A–B, D) cases. ***P< 0.001; **P< 0.01 using a paired *t-*test adjusted for clustering.

**Figure 2:**
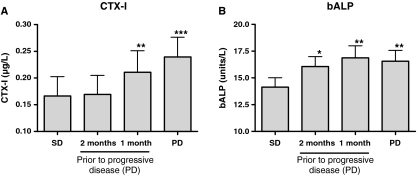
Changes in serum levels of CTX-I and bALP prior to progressive disease (PD). (A) CTX-I progressively increased reaching significance 1 month prior to first sign of progressive disease. (B) bALP reaches significance 2 months prior to PD, the increase reached a maximum 1 month prior to first sign of progressive disease. Results are shown as mean ± SEM of 40 cases (A–B). ***P< 0.001; **P< 0.01; *P< 0.05 comparing all values to value at stable disease (SD) using a paired *t-*test adjusted for clustering.

The bone resorption marker CTX-I showed a significant 44% increase when its level in a patient at first sign of PD was compared with its level in the same patient at nadir of the M-component or FLC (Fig. 1). The average increase in CTX-I became statistical significant 1 month prior to the detection of PD and subsequently increased further with time (Fig. 2), prior to that no consistent changes were observed (data not shown). Even though bALP levels were very low, with a mean level of 14.15 Units/L (normal range 15–41 Units/L), a significant 17 % increase was observed at first sign of PD (Fig. 1). bALP levels remained low during the entire period, and although the average increase became statistical significant 2 months prior to the detection of PD by conventional markers, values started to decline again before PD was reached (Fig. 2). No significant changes were observed in the bone degradation markers NTX-I or ICTP (Fig. 1). The increase in CTX-I and bALP could not be ascribed to deteriorating kidney function as serum creatinine remained stable in the observed period (*data not shown*).

### Correlation of markers of bone turnover with radiological evidence of progressive osteolysis

**Figure 3:**
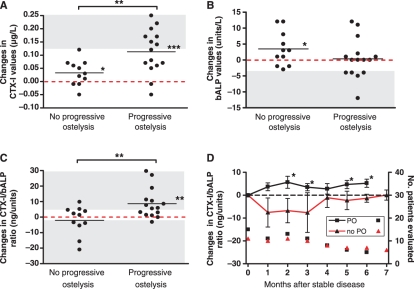
Changes in CTX-I and bALP in the individual patients stratified according to progression in osteolytic lesions. (A) Patient-specific CTX-I increased significantly in patients with progressive osteolysis, a much smaller although still significant increase was observed in patients with stable bone disease. The seven patients with the highest change in CTX-I all had progression in osteolysis. (B) bALP did not show a compensatory increase to CTX-I in patients with progressive osteolysis. The five patients with the greatest decline all had deteriorating bone status. In patients with stable bone disease, a small significant increase was observed in bALP thereby compensating for the likewise small increase in CTX-I. (C) The CTX-I/bALP ratio increased significantly in the patients with contemporary bone disease compared to patients with stable bone disease. Nine of ten patients with an increase over 5.2 had progressive osteolysis, whereas five of six patients with decreasing values had stable bone disease. (D) Development in the CTX-I/bALP ratio evaluated from stable disease and onward. Analysis was conducted until fewer than five datasets remained for evaluation. Patients with progressive osteolysis (PO) showed significantly increased values from the second month after stable disease. Patients with stable bone disease had a tendency to decreasing values although insignificant. Results are shown as patient-specific changes in bone markers as a mean of 11 and 15 cases, respectively (A–C). ***P< 0.001; **P< 0.01; *P< 0.05 using either an unpaired (A–C) or a paired (D) *t*-test adjusted for clustering.

CTX-I increased significantly both in patients with or without concomitant progression in bone disease. An increase of 0.11 μg/L was observed in patients with progressive osteolysis, whereas a significant smaller increase of 0.03 μg/L was observed in patients with no detectable progression in osteolysis. All patients with an observed CTX-I increase of more than 0.12 μg/L, regardless of starting levels, showed progressive osteolysis (Fig. 3A). The bone formation marker bALP remained highly suppressed in patients with progressive osteolysis, whereas a small increase was observed in patients with stable bone disease compensating for the small CTX-I increase. Furthermore, all cases where bALP decreased more that 3 units/L presented with progressive osteolysis (Fig. 3B) The different patterns of change of CTX-I and bALP levels in patients with and without radiological evidence of bone disease probably reflects increased uncoupling of bone remodeling in patients with progressive osteolysis. A CTX-I/bALP ratio was calculated for each patient to estimate the degree of uncoupling. At time of PD, the mean patient-specific ratio increased 8.7 ng/unit in patients with radiologically detected progression of bone disease. In contrast, the ratio was unchanged if no new bone lesions could be visualized. In case of a decreasing ratio, it was unlikely that a patient suffered from progressive osteolysis as it was only observed in 1 of 6 cases; sensitivity 93%. Contrasting this, ratios that increased more than 5.2 ng/units were a good indication of progressive osteolysis as this was observed in 9 of 10 cases; specificity 0.91% (Fig. 3C). Observing the ratio from SD and onward, there was a tendency for decreased values in the group with no detectable progression in bone disease, whereas the ratio on average increased significantly already 2 months after SD and remained positive in patients who later developed new osteolytic lesions (Fig. 3D). If ‘failure’ is defined as progression of bone disease, Cox regression analysis of the CTX-I/bALP ratio resulted in a hazard ratio of 1.077 (*P*< 0.05).

Surprisingly, two patients with radiologically detected progression of bone disease had decreased values of CTX-I. However, they also had decreased values of bALP which resulted in an increased CTX-I/bALP ratio in accordance with their progression of bone disease.

## Discussion

The average life expectancy of patients with MM has increased in recent years after introduction of several novel agents for treatment of patients with MM (37). During recent years, evidence has emerged suggesting that long-term treatment with potent bisphosphonates may cause ONJ. This has led to a more restrictive use of bisphosphonates. With prolonged survival of patients with MM because of use of novel agents, most patients will get to a point where the potential benefits and disadvantages of continued bisphosphonate treatment should be considered. Continued treatment will increase the risk of ONJ, whereas discontinued treatment will increase the risk of osteolysis and fractures. So far, there are no clinical data to guide us on an individual basis, neither about the optimal duration of initial bisphosphonate treatment nor on the risks and benefits of reinstituted bisphosphonate treatment at the time of progression. Recent guidelines recommend that radiographs should not be taken routinely, but only at tangible suspicion or symptoms of progression of osteolytic bone disease. However, with this approach, substantial and irreversible bone damage may occur before treatment with bone-protecting agents is initiated.

Several biochemical markers have been suggested in the past to be of value for evaluation of osteolytic bone disease in MM. However, they have only been tested either at diagnosis and used as prognostic markers or after initiation of new therapy. Our data are unique because they describe what happens before disease progression with or without progressive osteolysis occurs. This could only be done because we measured bone markers monthly in a large group of patients during a long period. Furthermore, previous studies have been conducted in strictly defined patient populations. The reason for this is probably that factors such as bisphosphonate treatment, disease stage, and to a lesser extent anti-myeloma treatment have a major impact on bone marker levels. We did likewise observations in our study, making analyses on absolute values obscure in this heterogeneous population. However, if we took into account patient-specific changes regardless of absolute values, we were able to identify a unique pattern. We discovered that patient-specific changes in CTX-I, bALP, and the ratio of CTX-I/bALP could distinguish between relapse with or without concomitant progression of bone disease and that changes in the CTX-I/bALP ratio increases in cases with new osteolytic lesions and otherwise remain unchanged. The Cox regression model reported an increased relative risk of progressive osteolysis to be 7.7% if the ratio increased with 1. In some patients, we observed that the ratio increased with up to 30.

It is noteworthy that skeletal surveys and/or CT scans were only performed in 26 of 40 cases. Skeletal imaging was not done routinely but only at suspicion of bone disease according to international guidelines. Moreover, it can be expected that some patients defined as having stable bone disease were miscategorized because of lack of sensitivity of the conventional radiography. If our findings are confirmed in a prospective setting where bone status is evaluated routinely in all patients using more sensitive methods e.g. CT scans, it can be expected that the group of patients without progression would be more homogeneous. Such a study will probably result in an even stronger separation of the CTX-I/bALP ratio when comparing patients with or without progressive bone disease.

That CTX-I and NTX-I may be used to monitor bone disease is a reasonable assumption. Collagen type I constitutes 90% of the organic bone matrix (38), and CTX-I and NTX-I are the most abundantly released products from collagen degradation (39). Furthermore, both markers are known to decline in patients responding to anti-myeloma treatment (32–34, 40, 41). Because of bisphosphonate treatment, both the values of CTX-I and NTX-I were suppressed. The CTX-I values, however, remained within detection range, whereas many NTX-I values were below the lower detection limit both at stable and progressive disease. It was probably because of this, we were unable to detect any increase in NTX-I. Thus, CTX-I seems to be a more robust marker for osteolytic activity than NTX-I in patients treated with bisphosphonates because of the CTX-I assays ability to detect changes even at low absolute levels.

ICTP is generated by other proteolytic enzymes compared to CTX-I and NTX-I (42) and has been shown to be of prognostic value in all ISS subgroups (27). In the present study, we found very high ICTP levels. ICTP, however, remained continuously elevated regardless of disease progression. Our findings are contradicted by two small studies that showed increasing ICTP values in patients with progressive disease (23, 43). However, both studies were carried out in bisphosphonate naïve patients. In conclusion, although ICTP is highly elevated in MM and yields valuable prognostic information when used at diagnosis, it appears less informative when used for continuous monitoring.

Bone disease in MM is not only because of an increased bone resorption but also caused by inhibition of bone formation. As expected, our patients had highly depressed bALP levels (mean 14.15; normal range 15–41). Even so, we were still able to detect an increase in the patients with stable bone disease compensating for an increase in CTX-I. The fact that osteoblast activity may increase to compensate for increased bone resorption in a well described phenomenon in early stage myeloma (44–46). Furthermore, it is interesting that no increase in bALP was observed in patients with progressive osteolysis. The importance of evaluating changes in both OC and OB function is highlighted by the fact that no patients in our study had progression in bone disease without showing either increasing CTX-I values or decreasing bALP values. Two patients with progression in bone disease had decreasing levels of both CTX-I and bALP. Thus, their progression was more likely caused by inhibition of the OB than by an activation of OC. New drugs targeting OB inhibitors are currently being investigated. Hypothetically, bone markers may help us to determine whether a patient will benefit most from treatment with bisphosphonates or e.g. antibodies against DKK-1.

In conclusion, we detected specific changes in bone markers prior to disease progression and progressive osteolysis. This was rendered possible because we did not look at absolute levels but at changes in the individual patient. The changes were consistent even in a very heterogeneous population. This gives our findings a high external validity, making it usable in everyday clinical life. Measurements of CTX-I and bALP, and the calculated CTX-I/bALP ratio, could become very important tools for monitoring myeloma bone disease and possibly guide the initiation of treatment with bisphosphonates or newer bone-protecting drugs before extensive bone destruction occurs (31, 41). However, prospective studies with preplanned bone imaging are needed to validate our findings and define relevant individual changes in the bone remodeling markers. Furthermore, randomized studies must prove if patient tailored treatment reduces progressive osteolysis.
